# The role of executive function abilities in interleaved vs. blocked learning of science concepts

**DOI:** 10.3389/fpsyg.2023.1199682

**Published:** 2023-11-06

**Authors:** Jimin Park, Keisha Varma, Sashank Varma

**Affiliations:** ^1^Department of Educational Psychology, University of Minnesota, Minneapolis, MN, United States; ^2^School of Interactive Computing and School of Psychology, Georgia Institute of Technology, Atlanta, GA, United States

**Keywords:** executive function, instructional sequencing, science instruction, interleaved learning, blocked learning, transfer

## Abstract

This study investigated the relative efficacy of interleaved versus blocked instruction and the role of executive function in governing learning from these instructional sequences. Eighth grade students learned about three rock concepts (igneous, sedimentary, metamorphic) and their attributes (origin, texture, composition). Consistent with prior studies and as predicted by current theoretical accounts, students who received interleaved instruction showed better memory (i.e., accuracy on true–false questions) when tested 2 weeks later, whereas those who received blocked instruction showed better memory when tested on the same day as instruction. Also consistent with prior studies and theoretical accounts, the blocked group showed greater transfer when tested after a retention interval, although this advantage was not significant. Critically, and as predicted, the shifting and inhibition executive function abilities were more predictive of learning from interleaved vs. blocked instruction. These findings lay the groundwork for future studies investigating the role of executive function in learning from different forms of instruction.

## 1. Introduction

**Figure 1:**
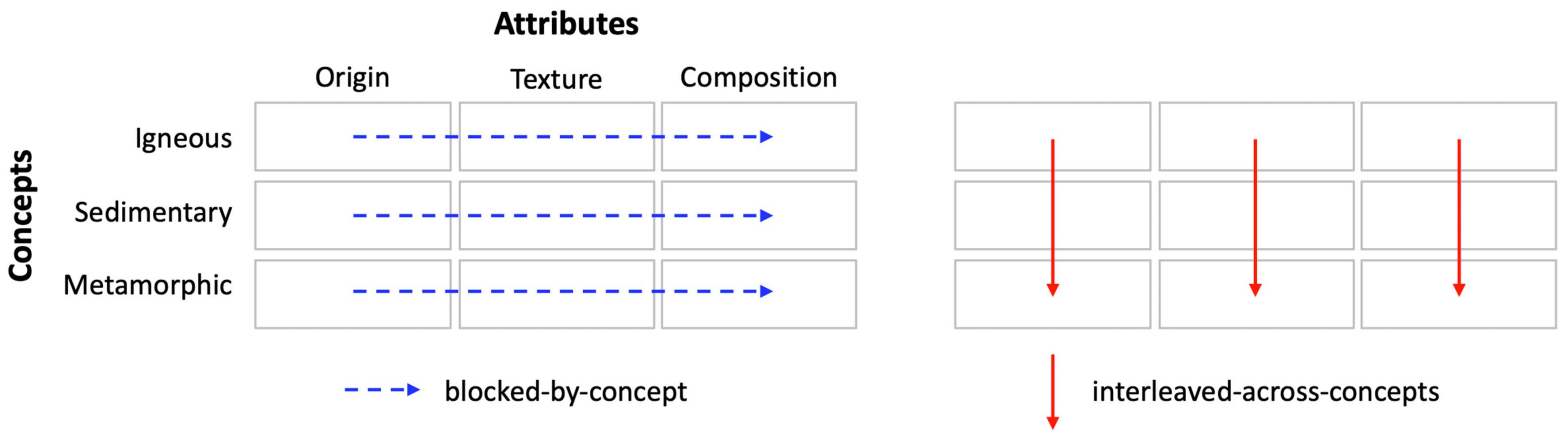
Visual depiction of the two instructional sequences.

Science concepts are defined and differentiated by their attributes. For example, for igneous rocks, their composition is determined by the amount of silica, whereas for metamorphic rocks, it is determined by how the grains are arranged. Instruction typically focuses on one concept at a time, presenting its attribute-values before moving on to the next concepts. For example, when learning about rocks, students might first learn about igneous rocks and their attributes (e.g., origin, texture, and composition), then learn about metamorphic rocks and their attributes, and so on. We refer to this organization as *blocked-by-concept* (i.e., interleaved-by-attribute). Blocked-by-concept instruction is ubiquitous in classrooms, perhaps because it aligns naturally with the conceptual capacities of school-age children (e.g., Sloutsky and Fisher, 2011). However, learning a concept is never so simple. Concepts have multiple representations, raising the question of how to sequence them. Here, we contrast blocked-by-concept sequencing with its inverse, *interleaved-across-concepts* (i.e., blocked by attribute). In this sequencing, students focus on one attribute at a time (e.g., origin) and learn its value for multiple concepts (e.g., igneous, sedimentary, and metamorphic rocks). They then shift to another attribute and learn its value for those concepts, and so on. These two instructional sequences are depicted in Figure 1.

The current study investigated whether blocked-by-concept instruction results in different levels of learning – memory for instructed information and transfer of this information to new problems – than interleaved-across-concepts instruction, and if so *when* and on *what* measures and for *whom*. Early studies of blocked-by-concept versus interleaved-across-concepts instruction were conducted in the laboratory with undergraduate participants, and educational recommendations were extrapolated from their findings (e.g., Rohrer and Taylor, 2007). Subsequent studies have been conducted in the classroom (e.g., Rau et al., 2013, 2014; Rohrer et al., 2014, 2020). Both the laboratory and classroom studies have focused primarily on teaching concepts from mathematics (e.g., fractions, algebra). Thus, it is important to investigate the relative benefits of block versus interleaved instruction for new domains. Here, we do so for science concepts (Eglington and Kang, 2017; Samani and Pan, 2021). Students received either blocked-by-concept instruction or interleaved-across-concepts instruction about three concepts (igneous, sedimentary, and metamorphic rocks) and three of their defining attributes (origin, texture, and composition). Learning was measured at two different time points, the day of instruction and two weeks later.

Most importantly, we investigated the role of executive function in governing learning from these instructional sequences. Executive function (EF) is a fundamental of cognitive abilities have been shown to predict learning and academic achievement in domains such as mathematics (van der Ven et al., 2012; Cragg and Gilmore, 2014; Lee and Bull, 2016; Nguyen et al., 2019), reading (Christopher et al., 2012; Georgiou and Das, 2016; Follmer, 2018; Barber et al., 2020), and science (St Clair-Thompson and Gathercole, 2006; Gropen et al., 2011; St. Clair-Thompson et al., 2012; Rhodes et al., 2013, 2016; Tolmie et al., 2016; Varma et al., 2018; Bauer and Booth, 2019; Mason and Zaccoletti, 2020; Kim et al., 2021). These findings leave open the question of whether EF abilities influence how well students learn from different types of instruction. In particular, which EF abilities are important for learning from interleaved-across-concepts instruction vs. blocked-by-concept instruction?

We evaluated three hypotheses. The first was that interleaved-across-concepts instruction would result in better memory performance on a long-term retention test but that blocked-by-concept instruction would result in better memory performance on the day of instruction. With respect to this hypothesis, the current study attempts to extend the findings of prior studies of mathematical concept acquisition (Rohrer and Taylor, 2007). The second hypothesis was that blocked-by-concept instruction would result in greater transfer at long-term retention. This follows from studies of analogical problem solving (Gick and Holyoak, 1983) and concept learning (Carvalho and Goldstone, 2015) showing that the comparison within a concept, made possible by blocked-by-concept instruction, results in greater noticing of similarities and greater transfer to new problems. The third, and critical, hypothesis concerned individual differences, specifically the role of executive function in learning from different instructional sequences. We predicted that the shifting and inhibition abilities would be particularly important for learning in the interleaved-across-concepts instruction, where students must rapidly switch their attention from one concept to another. This potentially corroborates the role of attention for sequencing instruction (Carvalho and Goldstone, 2017) and nuances a more general finding in the literature of a positive association between executive function skills and the science achievement scores of middle-school students (St Clair-Thompson and Gathercole, 2006; Varma et al., 2018).

### 1.1. Interleaved vs. blocked instruction

Although most conventional instruction is blocked, recent cognitive and educational psychology research has sometimes found better learning outcomes when instruction is interleaved – especially when learning is measured after a retention interval of several days or more (Rohrer and Taylor, 2007). The advantage of interleaved instruction has been shown in laboratory studies (Rohrer and Taylor, 2007; Kornell and Bjork, 2008; Eglington and Kang, 2017; Sana et al., 2017) and also in classroom studies (Taylor and Rohrer, 2010; Rau et al., 2013, 2014; Rohrer et al., 2014, 2015, 2020; Samani and Pan, 2021).

In an important study, Rohrer and Taylor (2007) examined undergraduate learning of geometric concepts under blocked-by-concept versus interleaved-across-concepts instruction. Participants learned to compute one attribute (volume) of four geometric concepts (wedge, spheroid, spherical cone, and half-cone solids). Participants experienced an instruction phase and a retention phase in a laboratory setting. During the instruction phase, participants received a tutorial on each solid and later solved 16 practice problems on each one. On the day of learning, participants in the blocked condition solved the practice problems more accurately than those in the interleaved condition. However, at the retention test given one week later, the interleaved group outperformed the blocked group (*d* = 1.34).^1^ This study showed the relative effectiveness of interleaved instruction when performance is measured after a retention interval of multiple days. However, it also showed that blocked instruction can be more effective in the short run, when considering performance during learning. Although an important demonstration, the laboratory nature of the study leaves open the question of whether interleaved instruction continues to be more effective when tested in science classroom contexts and with younger participants.

This pattern of superior performance during learning for blocked (i.e., massed) instruction and of superior performance after a retention interval of a few days or weeks for a different instructional condition – in this case, interleaved instruction – has been documented in many recent studies. Taylor and Rohrer (2010) found this pattern when the different instructional condition is interleaving. This pattern has also been shown when the different instructional condition is spacing (e.g., Karpicke and Roediger, 2007; Foster et al., 2019) or when it capitalizes on the testing effect (e.g., Roediger and Karpicke, 2006). We return to this pattern below when articulating the predictions of the current study.

To establish their educational relevance, it is important to replicate and extend laboratory findings in the classroom context. Rohrer and colleagues have investigated whether interleaved instruction is more effective than blocked instruction in classroom studies of fourth graders (Taylor and Rohrer, 2010) and seventh graders (Rohrer et al., 2014, 2020) learning mathematics concepts. For example, Rohrer et al. (2014) had 140 middle school students learn multiple algebraic concepts (i.e., solving linear equations, working with proportions, graphing linear equations, computing the slope of a line). The instruction phase spanned nine weeks and students received blocked-by-concept instruction and homework on some concepts and interleaved-across-concepts instruction and homework on others. Performance during the instruction phase was not measured because the teachers provided the solutions to students before scoring their homework. Two weeks after instruction ended, retention was measured by asking students to solve problems requiring the four concepts. Accuracy was almost twice as high when the underlying concepts had been learned from interleaved instruction (*d* = 1.05).

Eglington and Kang (2017) investigated whether the advantage of interleaved instruction holds for domains others than mathematics. They found that interleaved presentation of the diagrams of organic chemical compounds resulted in better memory for those structures when tested two days later than blocked presentation. Their study focused on the learning of pictures and diagrams, which illustrate the information conveyed in expository texts, but ultimately play a supporting role. Thus, the relative advantages of blocked versus interleaved instruction for learning science concepts remains a largely open question. We are aware of only one prior study that has directly addressed this question (Samani and Pan, 2021). College students enrolled in a physics course completed regular assignments where the problems were either blocked or interleaved by the concepts they required. On the assignments, students in the blocked condition performed better. However, on the surprise tests that were administered every several weeks, the pattern reversed, with students in the interleaved condition scoring better.

The current study addresses a number of the gaps identified above. It investigates whether interleaved-across-concepts instruction results in greater learning than blocked-by-concept instruction for science concepts studied by middle-school students. During the instruction phase, students experienced curricular materials comparable to those found in classroom textbooks and assessments. They learned about the different attributes of different rock concepts by working through a packet of textbook-like content. Each page included both expository text and pictures. Memory was measured on the day of instruction and again after a long-term retention interval. Following prior laboratory studies of mathematics concept learning, the prediction was that interleaved-across-concepts instruction would result in better memory for science concepts when measured two weeks after learning, but that blocked by-concept instruction would be superior when memory was tested on the day of instruction.

### 1.2. Transfer

Education is in the business of transfer. Its goal is learning where students are not just able to recognize or recall the information they have studied, but also able to apply that information to solve new kinds of unstudied problems. Educational psychology offers multiple theories of transfer (e.g., Bransford and Schwartz, 1999; Barnett and Ceci, 2002). Here, we focus on two approaches that predict greater transfer following blocked instruction versus interleaved instruction.

The first theory stems from the seminal study of transfer in the context of analogical problem solving by Gick and Holyoak (1983). They found that when participants were initially given multiple instances of a concept (versus just one instance), they were able to induce a new abstraction (i.e., schema) that covers these instances, and then were able to transfer this abstraction to understand new instances of the same concept. In this study, the materials were stories and problems from the Gestalt tradition that are arguably comparable in complexity to the content of science textbooks. This finding has been replicated and extended many times (e.g., Kurtz and Loewenstein, 2007; Minervino et al., 2017; Kalra et al., 2020). More generally, the power of comparing multiple instances for promoting subsequent transfer has been shown in contexts ranging from four-year-old children learning new concepts (Namy and Gentner, 2002) to middle-school students learning new algebra problem solving strategies (Rittle-Johnson and Star, 2011).

Converging support for the prediction that blocked instruction leads to greater transfer than interleaved instruction comes from the work of Carvalho and Goldstone. Their Sequential Attention Theory (SAT) proposes that blocked presentation focuses learners’ attention on the similarities between instances whereas interleaved presentation highlights the differences between them (Carvalho and Goldstone, 2014, 2015, 2017). Thus, blocked presentation should lead to better abstraction of new concepts or schemas, and ultimately to greater transfer to new problems. Carvalho and Goldstone (2021) found evidence for this prediction in a study science concept learning. They had undergraduates learned about four principles of scientific psychology (e.g., the availability heuristic) under blocked versus interleaved presentations. Experiments 1 and 1b found that participants in the blocked condition were better able to form an abstract understanding of the independent principles as measured by the quality of the definitions they wrote for them.

Consistent with Gick and Holyoak (1983) account of analogical problem solving and Carvalho and Goldstone’s (2015) SAT, we predicted an advantage for blocked-by-concept instruction over interleaved-across-concepts instruction on measures of transfer. These studies and the associated literature have typically measured transfer on the day of instruction. The more educationally relevant question is whether blocked instruction leads to greater transfer days or weeks later. To answer this question, we administered the transfer measure after a retention interval of two weeks.

### 1.3. Executive function abilities

It is critically important to investigate the fundamental cognitive abilities for learning from different instructional sequences. Some people may learn better from some instructional approaches than others. Here, we ask whether individual differences in executive function are relevant for predicting who will learn best from interleaved vs. blocked instruction. We adopt the Miyake et al. (2000) decomposition of executive function into three abilities:

- *shifting*, or switching between mental processes or representations;
- *inhibition*, or suppressing prepotent responses; and
- *updating*, or modifying the contents of working memory.

Individual differences in executive function have been shown to predict performance, learning, and achievement in academic domains such as mathematics (Cragg and Gilmore, 2014; Lee and Bull, 2016; Nguyen et al., 2019) and reading (Christopher et al., 2012; Follmer, 2018; Barber et al., 2020). However, only a handful of studies have examined their association to science learning and achievement. Kwon and Lawson (2000) investigated this relationship in a study of middle and high school students. They measured their executive function abilities, scientific reasoning skills, and learning of air pressure concepts following multiple weeks of instruction. The single best predictor of scientific reasoning skills was shifting ability as measured by the Wisconsin Card Sorting Task (WCST) – better than planning as measured by the Tower of London task, visuospatial ability as measured by the Grouped Embedded Figures task, and chronological age. Shifting ability was also the single best predictor of learning of air pressure concepts (i.e., pre-post gains). Varma et al. (2018) found that shifting as measured by the WCST and updating as measured by the digit span task were significant predictors of the scientific reasoning skills of middle school students. St Clair-Thompson and Gathercole (2006) found that inhibition as measured by the stop-signal task and updating as measured by the spatial span task predicted the science achievement scores of middle school students.

None of these studies investigated the role of executive function abilities in learning from different kinds of instruction. The current study addresses this gap. We predict that individual differences in the shifting and inhibition abilities in particular will be associated with learning from interleaved-across-concepts instruction. This instructional sequence focuses on one attribute at a time, showing its value across varying concepts. This should require inhibition to keep one’s attentional focus on the attribute of interest. This should also require shifting to switch across the varying concepts. (Note that switching from the previous concept to the next concept may also recruit inhibition to suppress the previous one.) Under this analysis, students with better shifting and inhibition abilities should be able to maximize their learning from interleaved-across-concepts instruction. By contrast, we predict no particular role for these executive functions in learning from blocked-by-concept instruction.

These predictions are consistent with theoretical proposals for why interleaving may be superior to blocking; this is the second reason we focus on executive function. The *discriminative contrast hypothesis* is that interleaved learning is superior to blocked learning because it promotes learning of the attributes that differentiate consecutively presented items (Kang and Pashler, 2012). Consistent with this reasoning is the SAT of Carvalho and Goldstone (2015) reviewed above. It proposes that during learning, people compare the current item with the previous item. For interleaved-across-concepts instruction, this will direct their *attention* to the different values that a focal attribute takes on across concepts.

The construct of attention that the SAT invokes is closely related to that of executive function, particularly inhibition ability (Engle and Kane, 2004; Diamond, 2013). No prior study has attempted to directly relate learning interleaved-across-concepts instruction to executive function. Thus, the prediction offered here – that shifting and inhibition abilities are associated learning in interleaved-across-concepts instruction – is theoretically important.

### 1.4. The current study

The current study evaluated the three hypotheses introduced above. The first was that interleaved-across-concepts instruction will result in better memory for information when measured a few weeks later. By contrast, blocked-by-concept instruction will result in better memory on the day of instruction itself. The second hypothesis was that blocked-by-concept instruction will better support the induction of abstractions than interleaved-across-concepts instruction, and thus result in a greater transfer when measured a few weeks later. The third hypothesis was that individual differences in two executive function abilities, shifting and inhibition, will be associated with learning in interleaved-across-concepts instruction, but not with learning in blocked-by-concept instruction.

This study was conducted with science concepts in a middle-school science classroom. Students experienced the instruction and retention phases as part of their normal classroom activities. The content was three rock concepts described along three attributes – information that was part of their standard curriculum. The intensity of the instruction and the assessments of learning (i.e., of memory for and transfer of the target information) were consistent with classroom norms, thus increasing the potential generalizability of the findings to typical classroom instruction.

## 2. Method

### 2.1. Participants

We used a quasi-experimental design, recruiting 115 eighth graders from a middle school serving a racially and ethnically diverse population in a metropolitan area in the Midwestern Unites States. We retrieved demographic data at the school level. The racial/ethnic breakdown was: 40% Hispanic, 30% Caucasian, 18% African American, 5% Asian, and 7% other. The gender breakdown was 52% female and 48% male. Participants were randomly assigned to receive either interleaved-across-concepts instruction or blocked-by-concept instruction at the class level. The study consisted of an instruction phase and a retention phase separated by two weeks. Complete data were available for *N* = 48 participants for the interleaved-across-concepts group and *N* = 46 for the blocked-by-concept group; the remaining participants completed the instruction phase but were not present in class during the retention phase. The experimental protocol was approved by the local IRB.

There was relatively little information to guide a power analysis because the current study focused on different academic content than prior classroom-based studies of interleaved-across-concepts learning (i.e., science vs. mathematics). The closest study is Rohrer et al. (2014), which shared with the current study a focus on a STEM domain, a target population of middle-school students, and a classroom-based (versus lab-based) setting. That study found a large (*d* = 1.05) advantage of interleaved instruction over blocked instruction. To have 85% power to detect the same-size effect in our study would require a sample of *n* = 18 for each instructional group.^2^ This estimate is likely too conservative because the Rohrer et al. (2014) study implemented interleaved instruction across multiple class periods distributed over multiple months, whereas our instructional phase occurred in a single class period. For this reason, we elected to make full use of our convenience sample of *N* = 94 participants with complete data.

### 2.2. Materials

#### 2.2.1. Learning materials

##### 2.2.1.1. Instructional study packet

**Figure 2:**
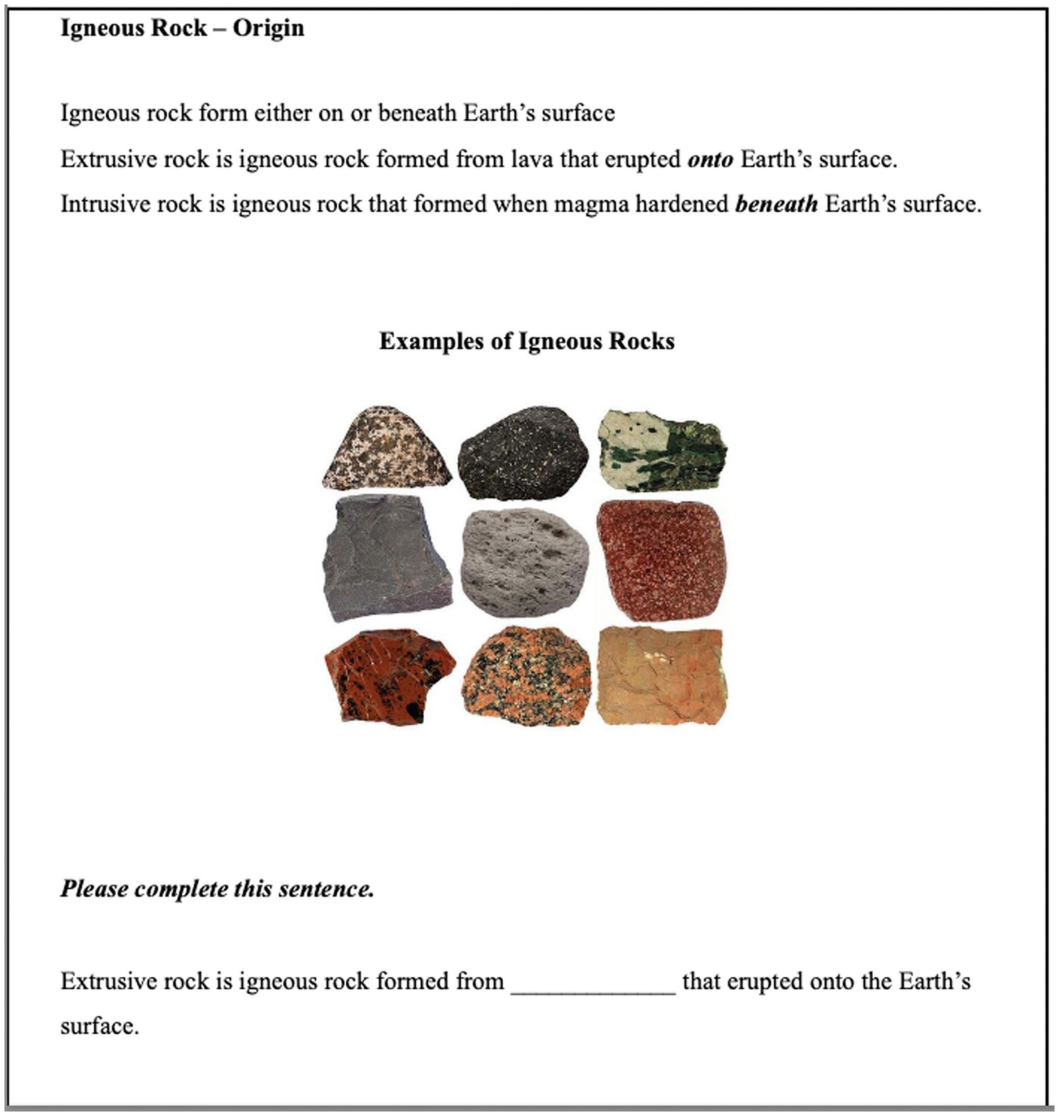
Sample page of the instructional study packet including the fill-in-the-blank question at the bottom.

The instructional study packet presented information about three rock concepts (igneous, sedimentary, metamorphic) and their attributes (origin, texture, composition). Each page of the nine-page packet provided a short paragraph of expository text about one attribute of one concept and one or more supporting images (Figure 2). Participants completed the packet by reading the information on each page and answering a fill-in-the-blank question at the bottom. The question was intended as a manipulation check to ensure that participants were engaging with the materials. (This check was important because participants were *not* pulled out and tested individually, but rather were run as part of their normal science class. Thus, it was important to verify that they attempted to understand the information in the packet.) The question was relatively easy, asking about information that was directly mentioned in the text.

The information was derived from the science textbook used in the classroom, “Middle Grade Science Earths Structure (Interactive Science)” (Buckley, 2011). This ensured that participating in the study aligned with students’ learning goals and their curriculum for the academic year. Participants only learned information from the packet; there was no accompanying lecture by the classroom teacher or the experimenter, and no additional seatwork or homework was assigned. The classroom teacher verified that the participants had no prior exposure to the material in their science classes.

The order of the pages in the packet differed for the two conditions; this was the critical manipulation. The blocked-by-concept group experienced each rock concept in turn, learning about each of its attribute values; see Figure 1. Thus, their instruction was blocked-by-concept (but interleaved-by-attribute). By contrast, the interleaved-across-concepts group experienced each attribute in turn, learning its value for each of the three rock concepts; see Figure 1. Thus, their instruction was interleaved-by-concept (but blocked-by-attribute). Participant were assigned to instructional conditions at the class level. This ensured that students were unaware that other students in the study experienced a different instructional sequence.

##### 2.2.1.2. Fill-in-the-blank test

Participants completed the same set of fill-in-the-blank questions twice. During the instruction phase, participants completed these questions one at a time, at the bottom of each page of the packet. See Figure 2 for a sample question. The answer to each question was stated explicitly on the page. Thus, it was possible for participants to answer the question by searching the text on the page, although they may of course have used different strategies (e.g., retrieving this information from their memory). Again, these questions were intended as manipulation checks to ensure that students were processing the information in the packet. They were *not* analyzed as measurements of learning.

During the retention phase, participants completed the same fill-in-the-blank questions from the instruction phase a second time, this time without the aid of the packet. For this administration, the nine questions were collected on a single-page and ordered randomly. See Table 1 for sample questions. This was the recall test. It was a true test of their memory because, during the retention phase, participants did not have access to the study packet when answering these questions. The dependent variable was the number of correct answers on these questions.

**Table 1:** Example questions from the learning (memory and transfer) tests.

Test	Example questions
Fill-in-the-Blank	The minerals in rock can change into other kinds of minerals as a result of very high heat or ____________. Intrusive rock has a ____________ texture. Clastic rocks are composed of shale and ____________.
True–false	Organic Rocks are composed of the remains of plants and animals. TRUE FALSE Breccia rocks have rounded edges. TRUE FALSE
Transfer	Statues, fortresses, bridges, and large public buildings are made up of shiny rocks. Based on what we learned about the texture of different types of rocks, which one is most appropriate for building these structures? Igneous rock Sedimentary rock Metamorphic rock ***Why did you make this choice?*** (please use complete sentences)

##### 2.2.1.3. True–false test

Participants responded to the same set of true–false statements twice. These questions were created by choosing one sentence from each of the nine pages of the packet and transforming it into a true–false statement. This statement was different than the information queried by the fill-in-the-blank question that appeared at the bottom of the page. See Table 1 for sample statements. Participants did not have access to the study packet while completing this test of recognition memory. The dependent variable was the number of correct answers, and this was computed for both phases.

During the instruction phase, the true–false test was given 20 min after participants had completed the packet. During the retention phase, the same test was given a second time, immediately after participants completed the fill-in-the-blank test.

##### 2.2.1.4. Transfer test

The transfer test asked three questions that went beyond the information given in the packet. Each required generalizing from what had been learned about the three attributes to make an inference about a fourth, unstudied attribute: the typical uses of a rock concept (e.g., to clad public buildings, which is true for igneous rocks). See Table 1 for a sample question. Each transfer item consisted of two parts. Participants first answered a multiple-choice question, and then they provided a short justification for why they chose their answer. The transfer test was only administered during the retention phase. The dependent variable was the number of correct answers to the multiple-choice questions. We did not analyze the justifications because the data were too sparse. Many students chose not to provide a justification, and the justifications that were provided were often only a sentence fragment.

Our aim was investigating how people apply information to new questions after weeks of interval. Thus, the transfer test was only administered during the retention phase. We will return to this possible limitation in the discussion.

#### 2.2.2. Executive function

##### 2.2.2.1. Local–global task

**Figure 3:**
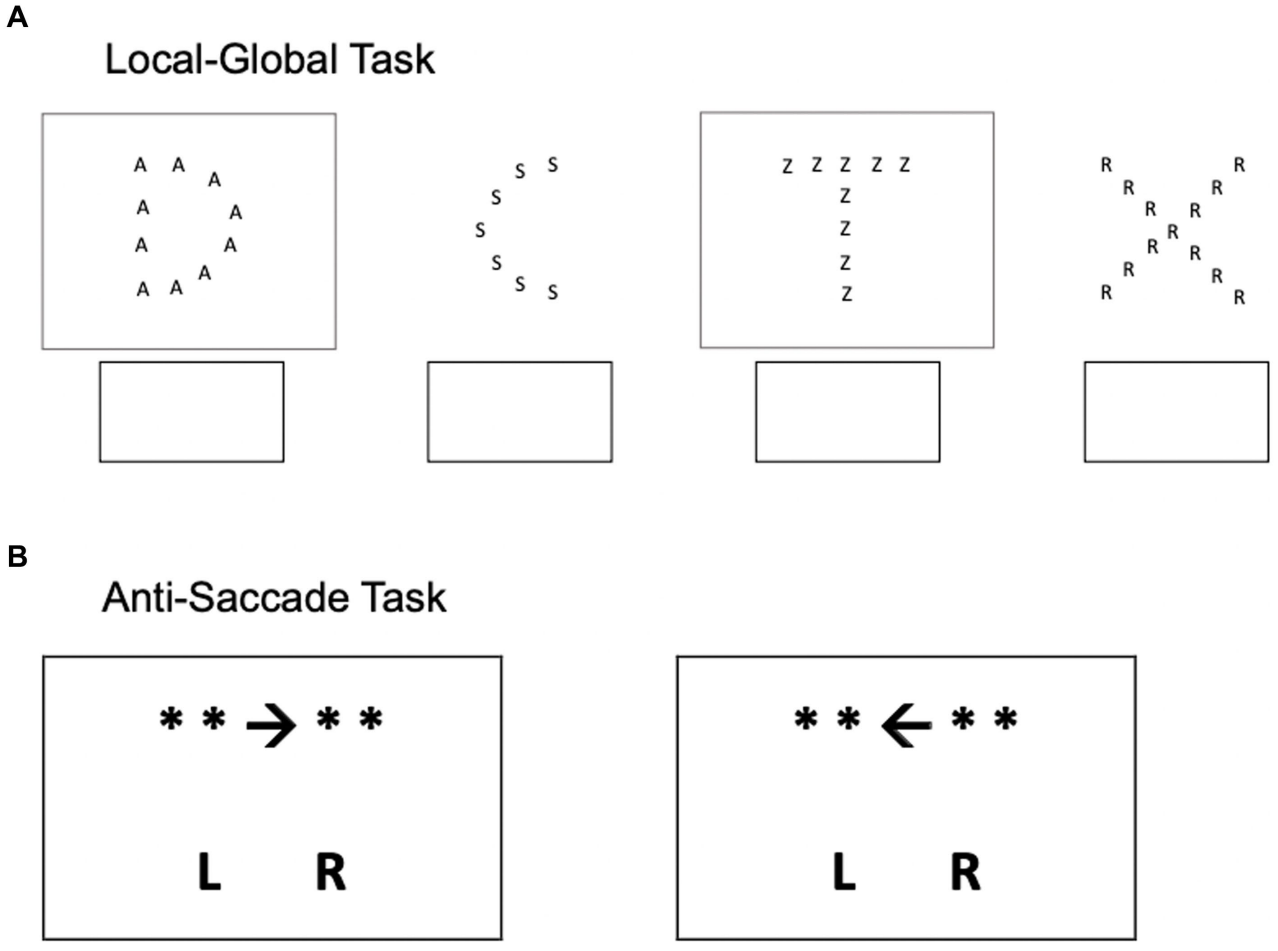
Example stimuli for the two executive function abilities, local-global task (shifting) and anti-saccade (inhibition).

We administered whole-class measures of two executive function abilities (Varma et al., 2017, 2018). Shifting was measured using a version of the local–global task adapted for group administration (Navon, 1977). See Figure 3A for example stimuli. Each was the form of a larger letter (e.g., “D”) composed of a number of copies of a different, smaller letter (e.g., “A”). If the letter was boxed, then participants had to indicate the larger letter; if it was unboxed, they had to indicate the smaller letter. The appearance of the box was randomized, and therefore, participants had to shift between the larger-letter and smaller-letter tasks on a trial-to-trial basis. There was a total of 90 stimuli. The dependent variable was the number of stimuli correctly completed in 2 min. Pilot testing confirmed that adults could not complete all 90 stimuli in 2 min, and indeed this was the case with the student participants of the current study. Thus, the task was implicitly speeded. We also explicitly instructed participants to complete as many stimuli as possible in the allotted time while maintaining high accuracy. Higher scores on this task indicate stronger shifting ability. The prediction is that for interleaved-across-concepts instruction, shifting should be positively associated with learning, i.e., memory and transfer.

##### 2.2.2.2. Anti-saccade task

Inhibition was measured using a version of the *anti-saccade* task adapted for group administration (Hallett, 1978; Roberts et al., 1994). See Figure 3B for sample stimuli. This task consisted of a sheet of 16 neutral stimuli and 16 interference stimuli randomly ordered in 8 rows of 4 stimuli each. For the neutral stimuli, participants had to indicate the direction of the arrow (e.g., “* * ← * *”) by circling “L(left)” or “R(right).” Interference stimuli were marked with a dot between the “L” and the “R” responses. For those stimuli, participants had to indicate the direction *opposite* that of the arrow. Thus, the interference stimuli required inhibiting the prepotent response of the arrow’s direction. Participants were explicitly instructed to complete the stimuli as quickly as possible without making errors. This time pressure was also signaled by the stopwatch project at the front of the classroom. After completing the sheet, participants inspected the stopwatch and recorded their completion time. The dependent variable was their response time (RT). Thus, a larger value indicates *weaker* inhibition ability. The prediction is that for interleaved-across-concepts instruction, this variable should be *negatively* associated with learning, i.e., memory and transfer.

### 2.3. Procedure

The experiment consisted of two phases, instruction and retention, separated by two weeks. During the instruction phase, each class of 20–25 students was randomly assigned to either the blocked-by-concept or interleaved-across-concepts instructional condition. Each participant received a packet where a total of nine pages were ordered according to their condition. Each page provided information about one of the three rocks’ attributes; see Figure 2 for an example page. Participants worked individually at their desks and at their own pace, sequentially reading each page and completing the fill-in-the-blank question at the bottom. Students required 10–15 min to complete the packet, which was then collected. Following a 20-min distractor task, participants then completed the true–false questions without access to the instructional packet.

During the retention phase, participants first completed the two executive function tasks: local–global measuring their shifting ability and anti-saccade measuring their inhibition ability. They then completed the fill-in-the-blank test and the true–false test. The questions for the fill-in-the-blank test were the questions that appeared at the bottom of the pages of their packet, randomly ordered. The questions for the true–false tests were the same as those experienced during the instruction phase, again ordered randomly. Participants completed all of the EF tasks and the content tests while working individually at their desks, requiring 20–25 min to do so. Importantly, they did not have access to the packet during the retention phase.

## 3. Results

The main goal of the current study was to investigate the role of executive function abilities in interleaved versus blocked instruction. We first evaluated the students’ learning gains and their transfer ability when they received interleaved versus blocked instruction. Then, we analyzed how individual differences in executive function abilities were correlated with the learning gains. All of the experimental materials are available in the Supplementary materials.

We first considered the manipulation check to ensure that participants actively engaged the study materials. These were the responses to the fill-in-the-blank questions that appeared at the bottom of each page of the packet, that were completed during the instruction phase. The answer to each question was stated explicitly on the page. Participants in the interleaved-across-concepts group answered 8.52 (*SD* = 1.05) of the 9 questions accurately on average. Those in the blocked-by-concept group answered 8.22 (*SD* = 1.55) questions accurately on average. The high level of performance indicates that participants in both instructional groups actively engaged the study materials. Note that the two groups did not differ on this measure, *t*(92) = 1.12, *p* = 0.27, *d* = 0.23.

### 3.1. Learning gains

#### 3.1.1. Memory

The first hypothesis was that the interleaved-across-concepts group would outperform the blocked-by-concept group when memory was tested at retention, but that the blocked-by-concept group would outperform the interleaved-across-concepts group when it was tested during instruction.

We first considered memory as measured by the fill-in-the-blank test. This test was only a learning measure at the retention phase, when it serves as a recall test. (Again, during the instruction phase, the same items served as the manipulation check.) We analyzed the fill-in-the-blank test data collected at retention using an independent samples *t* test. See Table 2 for the associated means and standard deviations. There was no difference in the performance of the two groups, *t*(92) = 0.30, *p* = 0.76, *d* = 0.06. Thus, contrary to the first hypothesis, interleaved-across-concepts instruction did *not* result in better memory than blocked-by-concept instruction when measured at the retention phase, at least using a recall test. We return to this failure in the discussion.

**Table 2:** Performance on the memory tests for the two instructional groups.

Test/Phase	Interleaved-across-concepts		Blocked-by-concept	
	*M*	*SD*	*M*	*SD*
*Fill-in-the-blank test*				
Retention phase	2.45	1.64	2.33	1.23
*True–false test*				
Instruction phase	5.65	1.33	6.13	1.41
Retention phase	5.65	1.56	5.22	1.46

For both tests, the maximum score is 9.

We next considered memory as measured by the true–false test, which serves as a recognition memory test. This test was administered to both instructional groups during both phases, and therefore supports a comprehensive evaluation of the first prediction. We analyzed these data in a repeated measures ANOVA with between-subject factor group (interleaved-across-concepts vs. blocked-by-concept instruction) and within-subject factor phase (instruction vs. retention). See Table 2 for the associated means and standard deviations. Not surprisingly, there was a main effect of phase, with participants performing better during the instruction phase than during the retention phase, *F*(1, 92) = 5.33, *p* = 0.023, η_p_^2^ = 0.055. There was no main effect of group (*F*(1, 92) = 0.015, *p* = 0.90, η_p_^2^ = 0.000). Critically, the group × phase interaction was significant, *F*(1, 92) = 5.57, *p* = 0.020, η_p_^2^ = 0.057. The performance of the interleaved-across-concepts group held steady from the instruction phase to the retention phase (*t*(47) = 0, *p* = 1, *d* = 0). By contrast, the performance of the blocked-by-concept group decreased significantly across this time interval, *t*(45) = 3.42, *p* < 0.01, *d* = 0.64. This interaction is consistent with our first hypothesis, that interleaved-across-concepts instruction results in more durable long-term retention than blocked-by-concept instruction.

### 3.2. Transfer

The second hypothesis was that blocked-by-concept instruction would result in greater abstraction of rock concepts, and would therefore produce greater transfer than interleaved-across-concepts instruction (e.g., Gick and Holyoak, 1983). Transfer performance was measured during the retention phase. We analyzed the transfer performance of the two instructional groups in an independent samples *t*-test. As predicted, the blocked-by-concept group (*M* = 1.39, *SD* = 1.04) scored higher than the interleaved-across-concepts group (*M* = 1.00, *SD* = 0.92). (Note that the maximum score was 3.) However, this difference not statistically significant, *t*(92) = −1.924, *p* = 0.058, *d* = 0.39. Thus, we found no support for the second hypothesis.

### 3.3. Executive function abilities

The third hypothesis was that two executive function abilities, shifting and inhibition, would be associated with learning for the interleaved-across-concepts group because these abilities are critical for rapidly switching between different concepts while learning about the same attribute. By contrast, no such association was expected for the blocked-by-concept group. To test this hypothesis, we examined the correlations between executive function abilities on one hand and performance on the learning (i.e., memory and transfer) tests on the other.

**Figure 4:**
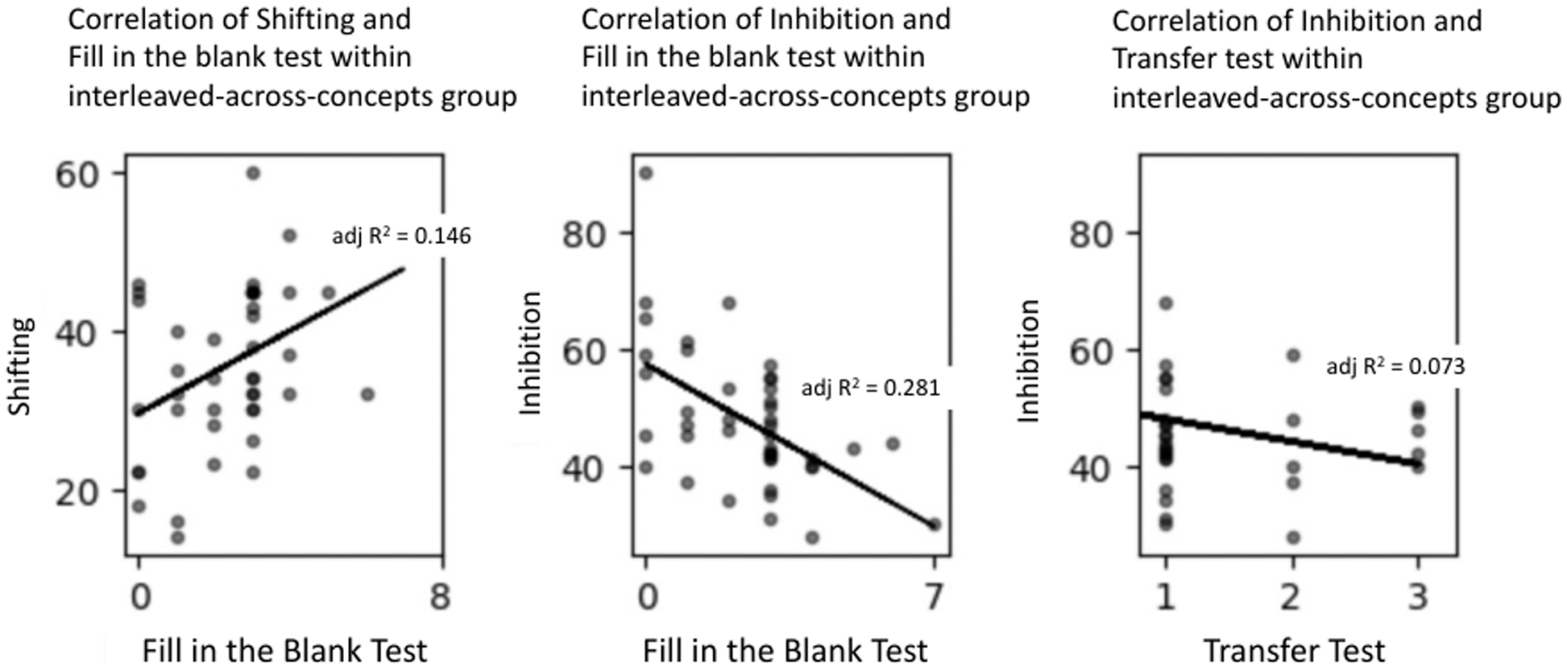
Correlations between executive function measures and learning measures for the interleaved-across-concepts group.

The descriptive statistics for the two executive functions measures for the two instructional groups are presented in Table 3. We first consider the correlations between these measures and the learning measures for the interleaved-across-concepts group. Note that there were missing data for five participants in this group (e.g., because of absences from class); thus, a total of 43 participants’ data were used in the following analyses. There were three significant correlations; see Table 4 and Figure 4. First, shifting skills were significantly associated with memory performance, *r*(41) = 0.408, *p* < 0.01. Second, inhibition skills were also significantly associated with their memory performance, *r*(41) = −0.546, *p* < 0.001. (This correlation is negative because the dependent variable for the inhibition measure is response time, and therefore *faster* response times are associated with *better* memory performance.) Interestingly, neither executive function measure was correlated with performance on the other memory measure, the true–false test, whether administered during the instruction phase or the retention phase. Finally, there was a significant correlation between inhibition ability and performance on the transfer test, *r*(41) = −0.308, *p* = 0.04.

**Table 3:** Descriptive statistics for the two executive function abilities for the two instructional groups.

Executive function	Interleaved-across-concepts				Blocked-by-concept			
	N*^a^*	*M*	*SD*	Range	N*^b^*	*M*	*SD*	Range
Shifting	43	47.70	11.73	29–90	41	34.68	9.20	9–54
Inhibition		35.77	10.41	14–60		49.95	13.71	30–90

a Because of missing data, 5 Interleaved-across-concepts participants were excluded.

b Because of missing data, 5 Blocked-by-concept participants were excluded.

**Table 4:** Correlations between executive function abilities and performance on the learning (memory and transfer) tests for the two instructional groups.

Test/Phase	Interleaved-across-concepts		Blocked-by-concept	
	Shifting	Inhibition	Shifting	Inhibition
*Fill-in-the-blank test*				
Retention phase	0.408*^a^*	−0.546*^b^*	0.361*^c^*	−0.256
*True–false test*				
Instruction phase	0.052	0.215	−0.009	−0.221
Retention phase	−0.003	−0.062	0.054	−0.071
*Transfer test*				
Retention phase	−0.009	−0.308*^d^*	0.265	−0.150

a *p* = 0.007.

b *p* < 0.001.

c *p* = 0.021.

d *p* = 0.046.

**Figure 5:**
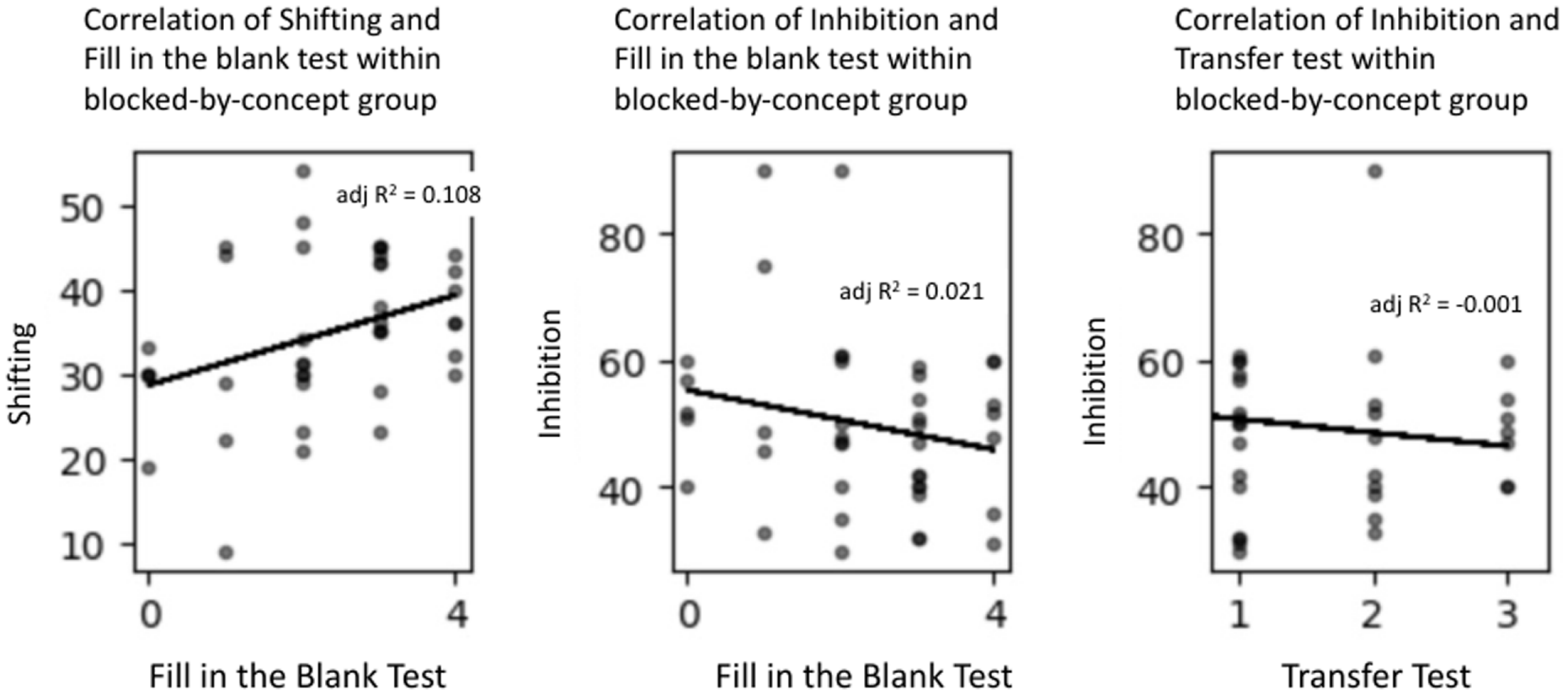
Correlations between executive function measures and learning measures for the blocked-by-concepts group.

For the blocked-by-concept group, we predicted no systematic associations between executive function abilities and performance on the memory and transfer tests. In fact, there was only one significant correlation; see Table 4 and Figure 5. Note that there were again missing data for five participants, leaving 41 in the final analyses. Only one executive function ability (i.e., shifting) was significantly correlated with performance on one memory test (i.e., the fill-in-the-blank test), *r*(39) = 0.361, *p* = 0.02.

## 4. Discussion

This study investigated the benefits of interleaved-across-concepts versus blocked-by-concept instruction for learning science concepts in a classroom setting. The first hypothesis was that students who received blocked instruction would show better memory when tested on the same day as instruction, whereas those who received interleaved instruction would show better memory when tested two weeks later. The current study found the predicted interaction for the true–false questions testing recognition memory. During the instruction phase, the blocked-by-concept group performed best. However, the pattern reversed during the retention phase, with the interleaved-across-concepts group performing best. Moreover, this advantage was not just statistically significant (*p* = 0.02), but the medium-to-large effect size (*d* = 0.64) suggests it may also be practically significant. This finding suggests that the interleaved instruction can be used to bolster learning of science concepts by middle school students in classroom contexts, consistent with a recent study of college students learning physics concepts (Samani and Pan, 2021). That said, there was no advantage of interleaved instruction on the second measure of memory: The two instructional groups performed comparably on the fill-in-the-blank questions during the retention phase. We return to this failure below.

The second hypothesis was that blocked-by-concept instruction would result in greater transfer during the retention phase. This follows from studies of analogical problem solving showing that comparing instances of the same concept promotes schema induction and transfer to new problems (Gick and Holyoak, 1983). It also follows from studies of concept learning showing the blocked presentation (vs. interleaved presentation) supports greater noticing of similarities within a concept and greater transfer to new problems (Carvalho and Goldstone, 2015; Carvalho and Goldstone, 2021). This prediction found no support (*p* = 0.058) in the current study and with a small to medium effect size (*d* = 0.39). The failure to reach statistical significance may have been due to the small number of questions (N = 3) on the transfer measure. Future studies should increase the number transfer questions and should also broaden the range of question types. This will increase the chances of detecting the predicted transfer effect if it indeed holds and will also enable a clearer evaluation of its practical significance.

The third hypothesis was that individual differences in executive function will be associated with learning in interleaved-across-concepts instruction, but not with learning in blocked-by-concept instruction. This prediction is novel within the literature. That interleaving recruits executive function is consistent with theories of why interleaving generally results in greater learning than blocking, such as the discriminative contrast hypothesis (Kang and Pashler, 2012) and the SAT (Carvalho and Goldstone, 2015). Both of these proposals invoke attentional mechanisms that are also associated with shifting and inhibition. That said, we are aware of no prior studies that directly investigated the role of executive function in learning from different instructional sequences. As predicted, for the interleaved-across-concepts group, both shifting ability and inhibition ability were correlated with performance on the fill-in-the-blank test during the retention phase. In addition, inhibition ability was correlated with performance on the transfer test during the retention phase. By contrast, and also as predicted, for the blocked-by-concept group, these abilities were generally uncorrelated with performance on the memory and transfer tests; the only exception was the association between shifting ability and performance on the fill-in-the-blank test during the retention phase. We discuss this pattern of findings in a greater detail below.

### 4.1. The complex relationship between executive functions and instructional sequences

Prior studies have found correlations between executive function abilities on one hand and scientific reasoning ability, science achievement scores, and learning of science concepts in classroom contexts (Kwon and Lawson, 2000; St Clair-Thompson and Gathercole, 2006; Varma et al., 2018). However, no prior study investigated the relationship between these abilities and learning from different instructional sequences. We predicted that shifting and inhibition are more important for learning from interleaved instruction than blocked instruction. Interleaved instruction focuses on one attribute at a time while the underlying concepts switch, requiring inhibition to maintain attentional focus and shifting to track the switching. By contrast, blocked instruction requires no particular role for these abilities. The findings, summarized in Table 4, were roughly consistent with this prediction. For the interleaved-across-concepts group, shifting and inhibition generally correlated with performance on one of the memory tests (i.e., the fill-in-the blank questions). However, they did not correlate with performance on the other memory test (i.e., true–false questions) at the retention phase. How should we understand this pattern of findings?

Existing theories of executive function offer little guidance in answering this question. Within the Miyake et al. (2000) framework adopted here, none of the three abilities are relevant for encoding or retrieving information from long-term memory. More recent versions of the theory have conjectured that the updating ability may be predictive of controlled retrieval from declarative memory (Miyake and Friedman, 2012; Friedman and Miyake, 2017). However, to our knowledge, no studies have directly tested this prediction.

However, a handful of studies have empirically investigated which memory tests are associated with which EF abilities. This work has mostly been done for tests of source memory working with developmental samples. Hala et al. (2016) found that the source memory performance of preschool children is predicted by their inhibition ability. Rajan et al. (2014) found that both source memory and fact recall are predicted by a composite EF variable that includes shifting, inhibition, and updating measures. Thus, there is some empirical evidence that EF is associated with performance on recall memory tests. However, we are aware of only one study that has investigated whether EF is associated with performance on a recognition memory test (Earhart and Roberts, 2014). This study found no such association, although it only measured one EF ability, inhibition.

The pattern across these studies is generally consistent with the one found in the current study. EF may be more relevant for recall tests, which require controlled retrieval processes, than for recognition tests, which might be driven by a more diffuse familiarity signal (Rotello et al., 2004). This differential pattern should be tested in future studies investigating the relationship between different EF abilities and different memory measures (i.e., recall vs. recognition) following learning of academic content.

### 4.2. Limitations and future directions

The findings of the current study should be understood with respect to its limitations. One limitation was the small sample size. There were 48 participants in the interleaved-across-concepts instruction group and 46 in the blocked-by-concept instruction group. This sample size was larger than in some prior laboratory studies [e.g., *N* = 18 across both groups in Exp. 2 of Rohrer and Taylor (2007)] but not others [e.g., *N* = 120 for each group in Exp. 1a of Kornell and Bjork (2008)]. With respect to classroom studies, the current sample size was larger than in some studies [e.g., *N* = 24 across both groups of Taylor and Rohrer (2010)] but not others [*N* = 126 for each group of Rohrer et al. (2014)]. Because the classroom context is noisier than the lab context, classroom studies typically require larger samples. Thus, the smaller sample size of the current study may have prevented detection of some of the predicted effects.

Another limitation was the short duration of the instructional intervention: there was only a single learning session. By comparison, the middle-school student participants of Rohrer et al. (2014) study completed 10 practice assignments spread over nearly three months. One reason for the smaller scale of the current study was that there have been many fewer studies of the efficacy of interleaved and spaced instruction (versus blocked instruction) for science concepts compared to mathematical concepts. We therefore started smaller, to gather initial information about the different instructional sequences for learning science concepts. Future studies should implement instructional interventions spanning multiple session, and over longer timescales.

Another limitation was that we only collected one measure of shifting and one of inhibition. This limitation was a consequence of the deliberate decision to conduct the study in a classroom context within the temporal constraints of normal class periods. The study was run during students’ science class periods, and thus for reasons of time, we were limited to only collecting one measure of shifting and one of inhibition. Future research should collect multiple measures of the target executive function abilities.

There were two other limitations caused by the time constraints. First, it is an open question whether the third executive function ability of Miyake et al. (2000) theory, updating, is associated with learning from interleaved (vs. blocked) instruction. Our theoretical analysis did not include a role for this ability, and so we chose not to measure it, again given the time constraints. Moreover, updating is related to working memory (WM), a cognitive ability that Sana and colleagues have explored with respect to learning from different instructional sequences (Sana et al., 2017, 2018). These studies have found mixed evidence for the role of WM in modulating learning. Sana et al. (2017) found that WM predicts learning from blocked instruction but not from interleaved instruction. However, the role of WM was of secondary concern in this study, and the authors wrote “given that the examination between WMC and study sequences in the current study is exploratory, we limit the interpretation of the results, which are speculative at best, and caution readers to do the same.” (p. 88). By contrast, the role of WM was the primary focus of Sana et al. (2018). Across four experiments, they found no evidence for this differential prediction. Future studies should investigate whether updating and WM are associated with learning from blocked versus interleaved instruction.

## Data availability statement

The original contributions presented in the study are included in the article/Supplementary material, further inquiries can be directed to the corresponding author.

## Ethics statement

The studies involving humans were approved by the University of Minnesota – Twin Cities. The studies were conducted in accordance with the local legislation and institutional requirements. Written informed consent for participation in this study was provided by the participants’ legal guardians/next of kin. Written informed consent was obtained from the individual(s) for the publication of any potentially identifiable images or data included in this article.

## Author contributions

All authors contributed to the conception and design of the study. JP, KV, and SV organized the database and wrote sections of the manuscript. JP and SV performed the statistical analysis. JP wrote the first draft of the manuscript. All authors contributed to manuscript revision, read, and approved the submitted version.

## Footnotes

^1^The computed power of this study was 0.73 given this value of *d* and the size of the interleaved group (*n* = 11) and the blocked group (*n* = 7).

^2^The required sample size was estimated using the pwr.t.test() function of the pwr R package. A 0.05 significance level was specified for an independent samples *t*-test comparing interleaved-across-concepts vs. blocked-by-concept instructional groups.

## References

[ref1] Barber A. T. Cartwright K. B. Stapleton L. M. Klauda S. L. Archer C. J. Smith P. (2020). Direct and indirect effects of executive functions, reading engagement, and higher order strategic processes in the reading comprehension of dual language learners and English monolinguals. Contemp. Educ. Psychol. 61:101848. doi: 10.1016/j.cedpsych.2020.101848

[ref2] Barnett S. M. Ceci S. J. (2002). When and where do we apply what we learn? A taxonomy for far transfer. Psychol. Bull. 128, 612–637. doi: 10.1037//0033-2909.128.4.612 12081085

[ref3] Bauer J.-R. Booth A. E. (2019). Exploring potential cognitive foundations of scientific literacy in preschoolers: causal reasoning and executive function. Early Child. Res. Q. 46, 275–284. doi: 10.1016/j.ecresq.2018.09.007

[ref4] Bransford J. D. Schwartz D. L. (1999). Rethinking transfer: a simple proposal with multiple implications. Rev. Res. Educ. 24, 61–100. doi: 10.2307/1167267

[ref5] Buckley D. (2011). Earth’s structure. Boston: Pearson.

[ref6] Carvalho P. F. Goldstone R. L. (2014). Putting category learning in order: category structure and temporal arrangement affect the benefit of interleaved over blocked study. Mem. Cogn. 42, 481–495. doi: 10.3758/s13421-013-0371-0, PMID: 24092426

[ref7] Carvalho P. F. Goldstone R. L. (2015). What you learn is more than what you see: what can sequencing effects tell us about inductive category learning? Front. Psychol. 6:505. doi: 10.3389/fpsyg.2015.00505 25983699 PMC4415402

[ref8] Carvalho P. F. Goldstone R. L. (2017). The sequence of study changes what information is attended to, encoded, and remembered during category learning. J. Exp. Psychol. Learn. Mem. Cogn. 43, 1699–1719. doi: 10.1037/xlm0000406, PMID: 28333507

[ref9] Carvalho P. F. Goldstone R. L. (2021). The most efficient sequence of study depends on the type of test. Appl. Cogn. Psychol. 35, 82–97. doi: 10.1002/acp.3740

[ref10] Christopher M. E. Miyake A. Keenan J. M. Pennington B. DeFries J. C. Wadsworth S. J.. (2012). Predicting word reading and comprehension with executive function and speed measures across development: a latent variable analysis. J. Exp. Psychol. Gen. 141, 470–488. doi: 10.1037/a0027375, PMID: 22352396 PMC3360115

[ref11] Cragg L. Gilmore C. (2014). Skills underlying mathematics: the role of executive function in the development of mathematics proficiency. Trends Neurosci. Educ. 3, 63–68. doi: 10.1016/j.tine.2013.12.001

[ref12] Diamond A. (2013). Executive functions. Annu. Rev. Psychol. 64, 135–168. doi: 10.1146/annurev-psych-113011-143750 23020641 PMC4084861

[ref13] Earhart B. Roberts K. P. (2014). The role of executive function in children’s source monitoring with varying retrieval strategies. Front. Psychol. 5:e405. doi: 10.3389/fpsyg.2014.00405, PMID: 24847302 PMC4021134

[ref14] Eglington L. G. Kang S. H. K. (2017). Interleaved presentation benefits science category learning. J. Appl. Res. Mem. Cogn. 6, 475–485. doi: 10.1016/j.jarmac.2017.07.005

[ref15] Engle R. W. Kane M. J. (2004). Executive attention, working memory capacity, and a two-factor theory of cognitive control. Psychol. Learn. Motiv. 44, 145–199. doi: 10.1016/S0079-7421(03)44005-X

[ref16] Follmer D. J. (2018). Executive function and reading comprehension: a meta-analytic review. Educ. Psychol. 53, 42–60. doi: 10.1080/00461520.2017.1309295

[ref17] Foster N. L. Mueller M. L. Was C. Rawson K. A. Dunlosky J. (2019). Why does interleaving improve math learning? The contributions of discriminative contrast and distributed practice. Mem. Cogn. 47, 1088–1101. doi: 10.3758/s13421-019-00918-4 30877483

[ref18] Friedman N. P. Miyake A. (2017). Unity and diversity of executive functions: individual differences as a window on cognitive structure. Cortex 86, 186–204. doi: 10.1016/j.cortex.2016.04.023, PMID: 27251123 PMC5104682

[ref19] Georgiou G. K. Das J. P. (2016). Direct and indirect effects of executive function on reading comprehension in young adults. J. Res. Read. 41, 243–258. doi: 10.1111/1467-9817.12091

[ref20] Gick M. L. Holyoak K. J. (1983). Schema induction and analogical transfer. Cogn. Psychol. 15, 1–38. doi: 10.1016/0010-0285(83)90002-6

[ref21] Gropen J. Clark-Chiarelli N. Hoisington C. Ehrlich S. B. (2011). The importance of executive function in early science education. Child Dev. Perspect. 5, 298–304. doi: 10.1111/j.1750-8606.2011.00201.x

[ref22] Hala S. Mc Kay L.-A. Brown A. B. Juan V. S. (2016). Source monitoring and executive function in 2.5- to 3-year-olds. J. Cogn. Dev. 17, 430–446. doi: 10.1080/15248372.2015.1058261

[ref23] Hallett P. E. (1978). Primary and secondary saccades to goals defined by instructions. Vis. Res. 18, 1279–1296. doi: 10.1016/0042-6989(78)90218-3, PMID: 726270

[ref24] Kalra P. B. Hubbard E. M. Matthews P. G. (2020). Taking the relational structure of fractions seriously: relational reasoning predicts fraction knowledge in elementary school children. Contemp. Educ. Psychol. 62:101896. doi: 10.1016/j.cedpsych.2020.101896, PMID: 32831458 PMC7442207

[ref25] Kang S. H. K. Pashler H. (2012). Learning painting styles: spacing is advantageous when it promotes discriminative contrast. Appl. Cogn. Psychol. 26, 97–103. doi: 10.1002/acp.1801

[ref26] Karpicke J. D. Roediger H. L. (2007). Expanding retrieval practice promotes short-term retention but equally spaced retrieval enhances long-term retention. J. Exp. Psychol. Learn. Mem. Cogn. 33, 704–719. doi: 10.1037/0278-7393.33.4.704, PMID: 17576148

[ref27] Kim M. H. Bousselot T. E. Ahmed S. F. (2021). Executive functions and science achievement during the five-to-seven-year shift. Dev. Psychol. 57, 2119–2133. doi: 10.1037/dev0001261, PMID: 34928663

[ref28] Kornell N. Bjork R. A. (2008). Learning concepts and categories. Psychol. Sci. 19, 585–592. doi: 10.1111/j.1467-9280.2008.02127.x 18578849

[ref29] Kurtz K. Loewenstein J. (2007). Converging on a new role for analogy in problem solving and retrieval: when two problems are better than one. Mem. Cogn. 35, 334–341. doi: 10.3758/bf03193454 17645174

[ref30] Kwon Y. J. Lawson A. E. (2000). Linking brain growth with the development of scientific reasoning ability and conceptual change during adolescence. J. Res. Sci. Teach. 37, 44–62. doi: 10.1002/(SICI)1098-2736(200001)37:1<44::AID-TEA4>3.0.CO;2-J

[ref31] Lee K. Bull R. (2016). Developmental changes in working memory, updating, and math achievement. J. Educ. Psychol. 108, 869–882. doi: 10.1037/edu0000090

[ref32] Mason L. Zaccoletti S. (2020). Inhibition and conceptual learning in science: a review of studies. Educ. Psychol. Rev. 33, 181–212. doi: 10.1007/s10648-020-09529-x

[ref33] Minervino R. A. Olguín V. Trench M. (2017). Promoting interdomain analogical transfer: when creating a problem helps to solve a problem. Mem. Cogn. 45, 221–232. doi: 10.3758/s13421-016-0655-2, PMID: 27718141

[ref34] Miyake A. Friedman N. P. (2012). The nature and organization of individual differences in executive functions. Curr. Dir. Psychol. Sci. 21, 8–14. doi: 10.1177/0963721411429458, PMID: 22773897 PMC3388901

[ref35] Miyake A. Friedman N. P. Emerson M. J. Witzki A. H. Howerter A. Wager T. D. (2000). The unity and diversity of executive functions and their contributions to complex “frontal lobe” tasks: a latent variable analysis. Cogn. Psychol. 41, 49–100. doi: 10.1006/cogp.1999.0734, PMID: 10945922

[ref36] Namy L. L. Gentner D. (2002). Making a silk purse out of two sow’s ears: young children’s use of comparison in category learning. J. Exp. Psychol. Gen. 131, 5–15. doi: 10.1037/0096-3445.131.1.5, PMID: 11900103

[ref37] Navon D. (1977). Forest before trees: the precedence of global features in visual perception. Cogn. Psychol. 9, 353–383. doi: 10.1016/0010-0285(77)90012-3

[ref38] Nguyen T. Duncan R. J. Bailey D. H. (2019). Theoretical and methodological implications of associations between executive function and mathematics in early childhood. Contemp. Educ. Psychol. 58, 276–287. doi: 10.1016/j.cedpsych.2019.04.002 31814657 PMC6897363

[ref39] Rajan V. Cuevas K. Bell M. A. (2014). The contribution of executive function to source memory development in early childhood. J. Cogn. Dev. 15, 304–324. doi: 10.1080/15248372.2013.763809, PMID: 24829540 PMC4016965

[ref40] Rau M. A. Aleven V. Rummel N. (2013). Interleaved practice in multi-dimensional learning tasks: which dimension should we interleave? Learn. Instr. 23, 98–114. doi: 10.1016/j.learninstruc.2012.07.003

[ref41] Rau M. A. Aleven V. Rummel N. Pardos Z. (2014). How should intelligent tutoring systems sequence multiple graphical representations of fractions? A multi-methods study. Int. J. Artif. Intell. Educ. 24, 125–161. doi: 10.1007/s40593-013-0011-7

[ref42] Rhodes S. M. Booth J. N. Campbell L. E. Blythe R. A. Wheate N. J. Delibegovic M. (2013). Evidence for a role of executive functions in learning biology. Infant Child Dev. 23, 67–83. doi: 10.1002/icd.1823

[ref43] Rhodes S. M. Booth J. N. Palmer L. E. Blythe R. A. Delibegovic M. Wheate N. J. (2016). Executive functions predict conceptual learning of science. Br. J. Dev. Psychol. 34, 261–275. doi: 10.1111/bjdp.12129 26751597

[ref44] Rittle-Johnson B. Star J. R. (2011). The power of comparison in learning and instruction: learning outcomes supported by different types of comparisons. Psychol. Learn. Motiv. Cogn. Educ. 55, 199–225. doi: 10.1016/b978-0-12-387691-1.00007-7

[ref45] Roberts R. J. Hager L. D. Heron C. (1994). Prefrontal cognitive processes: working memory and inhibition in the antisaccade task. J. Exp. Psychol. Gen. 123, 374–393. doi: 10.1037/0096-3445.123.4.374

[ref46] Roediger H. L. Karpicke J. D. (2006). Tested-enhanced learning: taking memory tests improves long-term retention. Psychol. Sci. 17, 249–255. doi: 10.1111/j.1467-9280.2006.01693.x 16507066

[ref47] Rohrer D. Dedrick R. F. Burgess K. (2014). The benefit of interleaved mathematics practice is not limited to superficially similar kinds of problems. Psychon. Bull. Rev. 21, 1323–1330. doi: 10.3758/s13423-014-0588-3 24578089

[ref48] Rohrer D. Dedrick R. F. Hartwig M. K. Cheung C. N. (2020). A randomized controlled trial of interleaved mathematics practice. J. Educ. Psychol. 112, 40–52. doi: 10.1037/edu0000367

[ref49] Rohrer D. Dedrick R. F. Stershic S. (2015). Interleaved practice improves mathematics learning. J. Educ. Psychol. 107, 900–908. doi: 10.1037/edu0000001

[ref50] Rohrer D. Taylor K. (2007). The shuffling of mathematics problems improves learning. Instr. Sci. 35, 481–498. doi: 10.1007/s11251-007-9015-8

[ref51] Rotello C. M. Macmillan N. A. Reeder J. A. (2004). Sum-difference theory of remembering and knowing: a two-dimensional signal-detection model. Psychol. Rev. 111, 588–616. doi: 10.1037/0033-295X.111.3.588, PMID: 15250777

[ref52] Samani J. Pan S. C. (2021). Interleaved practice enhances memory and problem-solving ability in undergraduate physics. Npj science of. Learning 6:e32. doi: 10.1038/s41539-021-00110-x, PMID: 34772951 PMC8589969

[ref53] Sana F. Yan V. X. Kim J. A. (2017). Study sequence matters for the inductive learning of cognitive concepts. J. Educ. Psychol. 109, 84–98. doi: 10.1037/edu0000119

[ref54] Sana F. Yan V. X. Kim J. A. Bjork E. L. Bjork R. A. (2018). Does working memory capacity moderate the interleaving benefit? J. Appl. Res. Mem. Cogn. 7, 361–369. doi: 10.1016/j.jarmac.2018.05.005

[ref55] Sloutsky V. M. Fisher A. V. (2011). The development of categorization. Psychol. Learn. Motiv. 54, 141–166. doi: 10.1016/b978-0-12-385527-5.00005-x

[ref56] St Clair-Thompson H. L. Gathercole S. E. (2006). Executive functions and achievements in school: shifting, updating, inhibition, and working memory. Q. J. Exp. Psychol. 59, 745–759. doi: 10.1080/17470210500162854, PMID: 16707360

[ref57] St. Clair-Thompson H. S. Overton T. Bugler M. (2012). Mental capacity and working memory in chemistry: algorithmic versus open-ended problem solving. Chem. Educ. Res. Pract. 13, 484–489. doi: 10.1039/c2rp20084h

[ref58] Taylor K. Rohrer D. (2010). The effects of interleaved practice. Appl. Cogn. Psychol. 24, 837–848. doi: 10.1002/acp.1598

[ref59] Tolmie A. K. Ghazali Z. Morris S. (2016). Children’s science learning: a core skills approach. Br. J. Educ. Psychol. 86, 481–497. doi: 10.1111/bjep.12119 27199279

[ref60] van der Ven S. H. G. Kroesbergen E. H. Boom J. Leseman P. P. M. (2012). The structure of executive functions in children: a closer examination of inhibition, shifting, and updating. Br. J. Dev. Psychol. 31, 70–87. doi: 10.1111/j.2044-835x.2012.02079.x, PMID: 23331107

[ref61] Varma K. Van Boekel M. Varma S. (2018). Middle school students’ approaches to reasoning about disconfirming evidence. J. Educ. Dev. Psychol. 8, 28–42. doi: 10.5539/jedp.v8n1p28

[ref62] Varma K. Varma S. Van Boekel M. Wang J. (2017). Studying individual differences in a middle school classroom context: considering research design, student experience, and teacher knowledge. Sage Research Methods Cases, Part 2. doi: 10.4135/9781473980150,

